# Extensively drug-resistant *Haemophilus influenzae* – emergence, epidemiology, risk factors, and regimen

**DOI:** 10.1186/s12866-020-01785-9

**Published:** 2020-04-28

**Authors:** Pei-Yi Su, Ay-Huey Huang, Chung-Hsu Lai, Hsiu-Fang Lin, Tsun-Mei Lin, Cheng-Hsun Ho

**Affiliations:** 1 Department of Laboratory Medicine, E-DA Hospital, Kaohsiung, Taiwan; 2 School of Medicine, College of Medicine, I-Shou University, Kaohsiung, Taiwan; 3 Division of Infectious Diseases, Department of Internal Medicine, E-Da Hospital, Kaohsiung, Taiwan; 4 Department of Medical Laboratory Science, College of Medicine, I-Shou University, No.8, Yida Road, Jiaosu Village, Yanchao District, Kaohsiung City, 82445 Taiwan

**Keywords:** *Haemophilus influenzae*, Antimicrobial susceptibility testing, Drug resistance, Cefotaxime

## Abstract

**Background:**

Concern about *Haemophilus influenzae* infection has been increasing over recent decades. Given the emergence of *H. influenzae* with severe drug resistance, we assessed the prevalence of as well as risk factors and potential therapies for extensively drug-resistant (XDR) *H. influenzae* infection in Taiwan.

**Results:**

In total, 2091 *H. influenzae* isolates with disk diffusion-based antibiotic susceptibility testing from 2007 to 2018 were enrolled. *H. influenzae* strains resistant to ampicillin, chloramphenicol, levofloxacin, and trimethoprim-sulfamethoxazole tended to be isolated from patient wards (≧41%), whereas those resistant to amoxicillin-clavulanate, cefotaxime, and cefuroxime were more likely to be isolated from intensive care units (approximately 50%). XDR *H. influenzae* was first identified in 2007, and its incidence did not significantly change thereafter. Overall prevalence of single, multiple, and extensively drug-resistant *H. influenzae* over 2007–2018 was 21.5% (*n* = 450), 26.6% (*n* = 557), and 2.5% (*n* = 52), respectively. A stepwise logistic regression analysis revealed that blood culture (odds ratio: 4.069, 95% confidence intervals: 1.339–12.365, *P* = 0.013) was an independent risk factor for XDR *H. influenzae* infection. No nosocomial transmission of XDR *H. influenzae* observed. Antibiotic susceptibility testing results demonstrated that cefotaxime was effective against 78.8% (*n* = 41) of the XDR strains.

**Conclusions:**

The presence of XDR *H. influenzae* strains was identified in Taiwan, and cefotaxime was efficacious against most of these strains.

## Background

*Haemophilus influenzae* is an opportunistic pathogen that can cause infections with various clinical symptoms, including otitis media, epiglottitis, sinusitis, and pneumonia, particularly in children, the elderly, and immunocompromised patients [1]. Transmission of *H. influenzae* occurs primarily through direct contact with respiratory droplets from pharyngeal carriers. Neonates may acquire infection by aspiration of amniotic fluids or by contact with genital tract secretions containing living bacteria [2]. Without early and effective treatments, *H. influenzae* infection may result in life-threatening complications, such as bacteremia and meningitis, particularly in those infected with type b strains. Bacteremia can lead to amputation of limbs. Moreover, up to 30% of adult patients who survive meningitis suffer permanent hearing loss or other long-term neurological sequelae [3]. Approximately 5% of invasive *H. influenzae* infections in children are fatal.

Although the vaccination plan against the type b strain has been promoted globally, *H. influenzae* remains a formidable pathogen in Taiwan because of the late introduction of the vaccine, an unsatisfactory implementation rate, and infections caused by non-type b or non-typeable strains. With regard to treatment, ampicillin and first-line cephalosporins are becoming increasingly less efficacious against *H. influenzae* due to the development of various drug resistant mechanisms, such as β-lactamase activity and modified bacterial penicillin-binding proteins [4–6]. Second or third-generation cephalosporins or quinolones have become more favorable options for the treatment of *H. influenzae* infection [7]. However, physicians should more cautiously select antibiotics because of the thriving *H. influenzae* that is non-susceptible to amoxicillin-clavulanate, fluoroquinolones, second or third-generation cephalosporins, and macrolides [7–12]; hence, carbapenems or combination therapies might be empirically considered if appropriate.

Multiple drug-resistant (MDR) *H. influenzae*, which is generally defined as non-susceptibility to at least one agent in three or more antimicrobial categories [13], was first reported in West Germany in 1980 [14]. In that report, two strains were non-susceptible to ampicillin, chloramphenicol, and tetracycline. The emergence of MDR *H. influenzae* has aroused the widespread concern of government health agencies, medical communities, and researchers worldwide. Much effort has been taken to reveal risk factors, adequate management control, prevention, and underlying mechanisms of acquired MDR activity [15–17]. Despite growing evidence of the identification of MDR *H. influenzae* strains in different countries [18–21], no study has demonstrated extensively drug-resistant (XDR) *H. influenzae*, defined as non-susceptibility to at least one agent in all but two or fewer antimicrobial categories [13]. Herein, we reported the emergence of XDR *H. influenzae* in South Taiwan. Furthermore, risk factors and antimicrobial susceptibilities of XDR *H. influenzae* were characterized.

## Results

### Epidemiology of drug non-susceptibility in *H. influenzae*

**Fig. 1:**
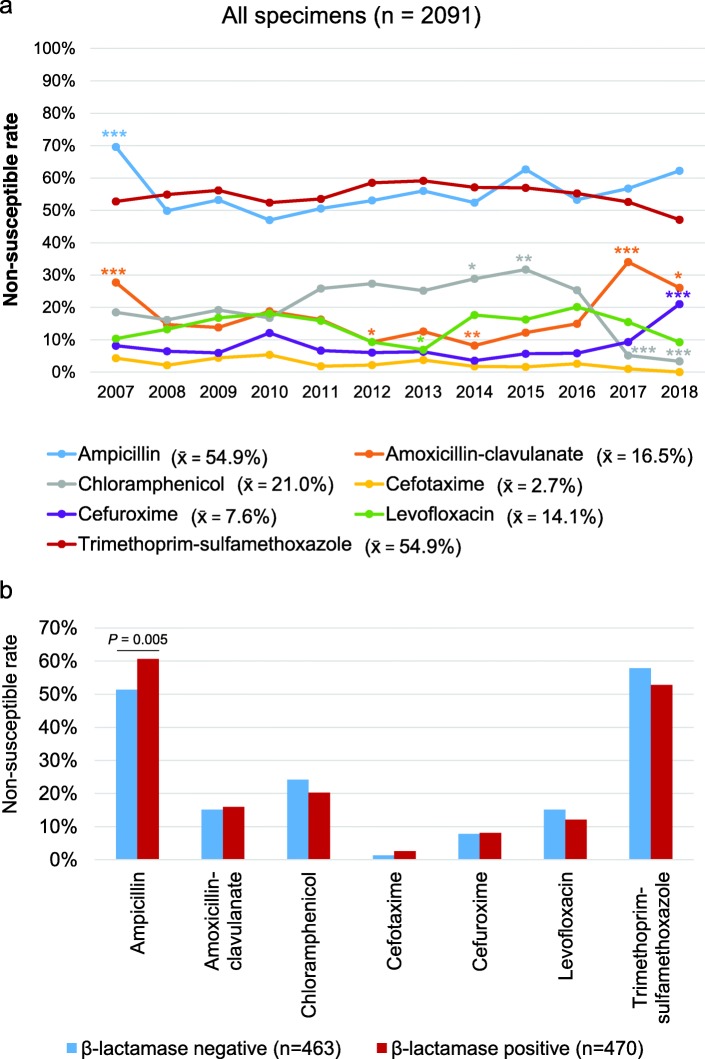
Drug non-susceptibility in *Haemophilus influenzae*. **a** Incidences of drug non-susceptibility to different antimicrobial agents in *H. influenzae* isolates from 2007 to 2018 (*n* = 2091) are shown. Fisher’s exact tests or Pearson Chi-square tests were used to assess the significance of the non-susceptible rate of each drug in each year when compared to the overall mean value. *P <  0.05, **P <  0.01, ***P <  0.001. x̄, mean value. **b** Comparisons of drug non-susceptibility rates in β-lactamase-positive and β-lactamase-negative *H. influenzae* isolates. *P*-values are obtained from Fisher’s exact tests

Characteristics of the patients and specimens (*n* = 2091) from which *H. influenzae* isolates were obtained are shown in Table 1. Most of the specimens were taken from respiratory tracts (*n* = 1915; 91.6%) including sputa, bronchial brushings, bronchial washings, and bronchoalveolar lavages. Forty-five (2.1%) isolates were obtained from blood cultures and 131 (6.3%) from wounds, abscesses, or body fluids. More than half of the *H. influenzae* isolates were non-susceptible to ampicillin or trimethoprim-sulfamethoxazole (Fig. 1a). In addition, 7.6, 2.7, and 14.1% of *H. influenzae* isolates were non-susceptible to cefuroxime, cefotaxime, and levofloxacin, respectively. Remarkably high resistance rates of *H. influenzae* to ampicillin was recorded in 2007 (69.6%) and to amoxicillin-clavulanate in 2007 (27.7%) and 2017 (34.0%). The non-susceptible rate of *H. influenzae* to chloramphenicol or cephems was less than 30%, except for chloramphenicol in 2015 and cefuroxime in 2017. Trends of drug non-susceptibility in different types of specimens are shown in Additional file 1: Figure S1. β-lactamase activity was screened in 933 *H. influenzae* isolates and significantly increased the drug non-susceptibility of ampicillin (*P* = 0.05) but not other antimicrobial agents (Fig. 1b). The demographics of the patients had almost no effect on the drug susceptibility of *H. influenzae* (Additional file 2: Figure S2).

**Table 1:** Characteristics of patients and specimens with *Haemophilus influenzae*

Year	Number	Patient gender (male: female)	Patient age	Specimen type (Respiratory, blood, others)	Specimen source (OPD, ER, Wards, ICUs)
2007	184	115: 69	75.7 ± 18.6	173, 5, 6	14, 8, 102, 60
2008	279	192: 87	73.0 ± 19.3	262, 3, 14	23, 23, 159, 74
2009	203	143: 60	68.0 ± 21.5	188, 2, 13	19, 21, 106, 57
2010	149	104: 45	69.4 ± 21.5	133, 1, 15	26, 20, 62, 41
2011	271	187: 84	63.8 ± 24.4	218, 6, 47	77, 42, 96, 56
2012	183	135: 48	64.2 ± 21.9	146, 2, 35	38, 36, 75, 34
2013	159	96: 63	61.7 ± 22.3	134, 6, 19	48, 33, 45, 33
2014	170	126: 44	66.0 ± 21.0	144, 5, 21	39, 32, 69, 30
2015	123	88: 35	67.0 ± 20.4	109, 3, 11	21, 18, 54, 30
2016	154	97: 57	62.8 ± 19.0	125, 5, 24	48, 13, 62, 13
2017	97	69: 28	61.7 ± 18.4	86, 3, 8	18, 8, 40, 31
2018	119	84: 35	60.9 ± 21.5	99, 4, 16	36, 11, 42, 30
Total	2091	1436: 655	66.8 ± 21.5	1915, 45, 131	407, 265, 912, 507

Age is shown as mean ± standard deviation. Specimens other than those from respiratory tracts and blood include wound, pus, abscess, body fluids, and tissues. *Abbreviations*: *ER* emergency room, *ICU* intensive care unit, *OPD* outpatient department

### Drug non-susceptibility of specimens from different origins

**Fig. 2:**
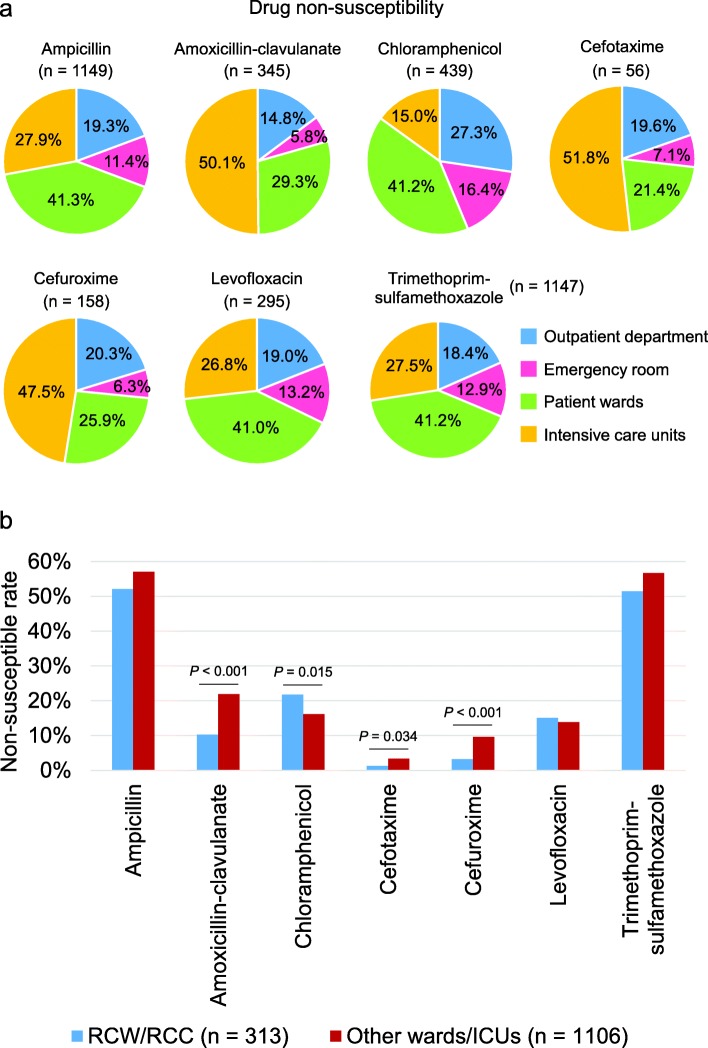
Drug non-susceptibility of *Haemophilus influenzae* from different origins. **a** Percentages of drug non-susceptible *H. influenzae* in specimens from different departments are shown. **b** Comparisons of drug non-susceptibility rates in hospitalized patients between respiratory care wards/center (*n* = 313) and other units (*n* = 1106) are shown in column graphs. *P*-values are obtained from Chi-square tests

*H. influenzae* strains that were isolated from specimens collected in the emergency room had the lowest non-susceptibility frequency to antimicrobial agents, except chloramphenicol (Fig. 2a). Patient wards were the major origin of *H. influenzae* isolates that were non-susceptible to ampicillin, chloramphenicol, levofloxacin, and trimethoprim-sulfamethoxazole, whereas intensive care units were the main origin of isolates non-susceptible to amoxicillin-clavulanate, cefuroxime, and cefotaxime. Notably, *H. influenzae* strains isolated from respiratory care wards or respiratory care centers were more susceptible to amoxicillin-clavulanate, cefotaxime, and cefuroxime but more often non-susceptible to chloramphenicol compared with other wards or intensive care units (Fig. 2b).

### MDR and XDR *H. influenzae*

**Fig. 3:**
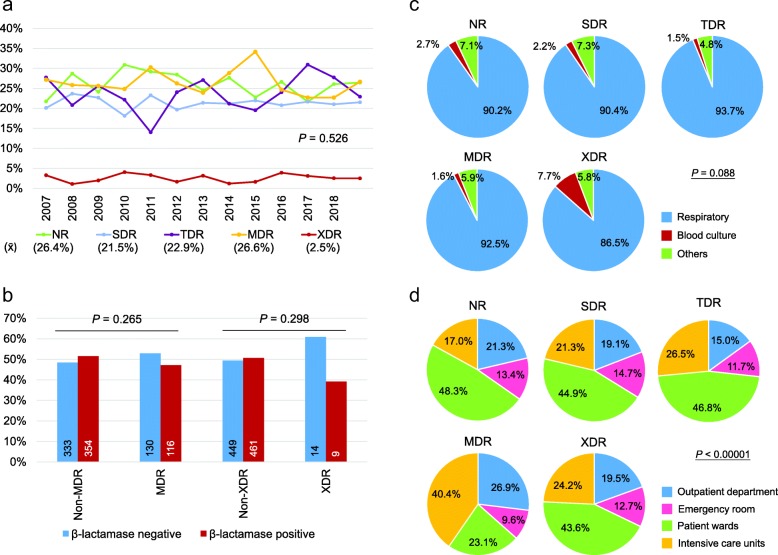
Drug resistant status of *Haemophilus influenzae*. (**a**) Trends of different *H. influenzae* drug resistant status from 2007 to 2018 are shown. (**b**) β-lactamase-positive rates in MDR, non-MDR, XDR, and non-XDR *H. influenzae* isolates are shown. The number of each group is shown in the bar. *P*-values are obtained from Pearson Chi-square tests. Proportions of different drug resistant status under (**c**) different specimen types and (**d**) different specimen sources are shown. Pearson Chi-square tests were used to assess the significance of each analysis. MDR, multiple drug-resistance; NR, no resistance; SDR, single drug-resistance; TDR, two drug-resistance; x̄, mean value; XDR, extensive drug-resistance

XDR *H. influenzae* strains were isolated from samples collected as far back as 2007. Incidences of single-drug resistance, MDR, and XDR *H. influenzae* from 2007 to 2018 were 21.5% (450/2091), 26.6% (557/2091), and 2.5% (52/2091), respectively (Fig. 3a). The drug resistance status remained consistent year by year (Fig. 3a, *P* = 0.526) and was not affected by the demography of the patients (Additional file 3: Figure S3A). Overall, MDR strains ranged from approximately 15 to 30% and XDR strains were all below 5%. β-lactamase activity did not correlate with the incidence of MDR (*P* = 0.265) or XDR (*P* = 0.298) *H. influenzae* (Fig. 3b). The drug non-susceptibility panels of MDR and XDR *H. influenzae* strains are shown in Table 2.

**Table 2:** Panels of drug non-susceptibility in multiple drug resistant (MDR) and extensive drug resistant (XDR) *Haemophilus influenzae* isolates

	Penicillin	β-lactam combination agent	Phenicol	Cephem		Fluoroquinolone	Folate pathway antagonist	
	Ampicillin	Amoxicillin-clavulanate	Chloramphenicol	Cefotaxime	Cefuroxime	Levofloxacin	Trimethoprim-sulfamethoxazole	n
MDR	V		V				V	190
	V		V			V	V	83
	V	V					V	81
	V	V	V				V	43
	V	V			V		V	26
	V	V				V	V	25
	V	V			V			24
	V					V	V	14
	V	V		V	V		V	14
	V	V		V	V			7
			V			V	V	6
	V	V		V				6
	V				V	V	V	6
	V		V			V		5
	V	V		V			V	5
	V	V	V					4
					V	V	V	3
	V		V		V			3
	V				V		V	3
	V		V		V		V	3
	V			V			V	2
	V	V				V		2
	V				V	V		1
	V	V	V	V	V			1
XDR	V	V			V	V	V	12
	V	V	V		V		V	11
	V	V	V			V	V	9
	V	V	V		V	V	V	8
	V	V		V	V	V	V	6
	V	V	V	V	V		V	3
	V		V		V	V	V	1
	V	V	V	V			V	1
	V	V		V		V	V	1

Multiple drug resistant (MDR) is defined as acquired non-susceptibility to at least one agent in three of five antimicrobial categories or non-susceptibility to at least one agent in four of five antimicrobial categories but less than five antimicrobial agents. Extensive drug resistant (XDR) is defined as acquired non-susceptibility to at least one agent in four of five antimicrobial categories and at least five antimicrobial agents

The type of specimen was not associated with the drug resistant status of *H. influenzae* (Fig. 3c). However, 7.7% of XDR strains were isolated from blood cultures. Regarding the origin of the specimen, MDR strains were more likely to be isolated from intensive care units (Fig. 3d). Neither MDR nor XDR *H. influenzae* strains were linked to hospital-acquired infections (Additional file 3: Figure S3B). Characteristics of the patients and specimens with XDR *H. influenzae* are shown in Additional file 4: Table S1. Among these 52 XDR *H. influenzae* strains, six were susceptible to chloramphenicol only and three to levofloxacin only (Table 3). Moreover, one strain was susceptible to cefuroxime and chloramphenicol and one was susceptible to cefuroxime and levofloxacin. The other 41 strains (78.8%) were either susceptible to cefotaxime only or cefotaxime together with an additional antimicrobial agent. These results suggest that cefotaxime is a potent antimicrobial agent for the management of XDR *H. influenzae* infection.

**Table 3:** Cross table of antimicrobial agent susceptibility in 52 extensive drug resistant *Haemophilus influenzae* isolates

	AM	AMC	C	CTX	CXM	LVX	SXT
AM	0 (0.0%)						
AMC	0 (0.0%)	0 (0.0%)					
C	0 (0.0%)	0 (0.0%)	6 (11.5%)				
CTX	0 (0.0%)	1 (1.9%)	12 (23.1%)	8 (15.4%)			
CXM	0 (0.0%)	0 (0.0%)	1 (1.9%)	9 (17.3%)	0 (0.0%)		
LVX	0 (0.0%)	0 (0.0%)	0 (0.0%)	11 (21.2%)	1 (1.9%)	3 (5.8%)	
SXT	0 (0.0%)	0 (0.0%)	0 (0.0%)	0 (0.0%)	0 (0.0%)	0 (0.0%)	0 (0.0%)

Data are shown as numbers (%). *AM* ampicillin, *AMC* amoxicillin-clavulanate, *C* chloramphenicol, *CTX* cefotaxime, *CXM* cefuroxime, *LVX* levofloxacin, *SXT* trimethoprim-sulfamethoxazole

### Clinical relevance of MDR and XDR *H. influenzae*

Stepwise logistic regression analyses revealed that MDR *H. influenzae* strains were less likely to be isolated from male patients (odds ratio [OR]: 0.769, 95% confidence intervals [CI]: 0.624–0.946; *P* = 0.013) (Table 4). Furthermore, a relationship between MDR *H. influenzae* and intensive care units (OR: 1.410, 95% CI: 1.094–1.818; *P* = 0.008) was noted. XDR *H. influenzae* was more likely to be isolated from blood cultures (OR: 4.069, 95% CI: 1.339–12.365; *P* = 0.013). β-lactamase activity was not associated with MDR (OR: 0.985, 95% CI: 0.875–1.109; *P* = 0.802) or XDR (OR: 0.936, 95% CI: 0.671–1.304; *P* = 0.694) *H. influenzae*. Furthermore, MDR (OR: 1.408, 95% CI: 0.789–1.392; *P* = 0.747) and XDR (OR: 0.394, 95% CI: 0.122–1.274; *P* = 0.120) *H. influenzae* strains were not transmitted via hospital-acquired modes of infection.

**Table 4:** Logistic regression analyses of factors associated with multiple drug resistance or extensive drug resistance in *Haemophilus influenzae*

Variable	Multiple drug resistant				Extensive drug resistant			
	Univariate		Multivariate		Univariate		Multivariate	
	Odds ratio (95% CI)	*P*	Odds ratio (95% CI)	*P*	Odds ratio (95% CI)	*P*	Odds ratio (95% CI)	*P*
***Demography***								
Year	0.995 (0.966–1.025)	0.735			1.019 (0.938–1.106)	0.664		
Age	0.997 (0.993–1.002)	0.244			0.994 (0.982–1.006)	0.332		
Gender (Male = 1, Female = 0)	0.786 (0.640–0.966)	0.022	0.769 (0.624–0.946)	0.013	1.128 (0.615–2.071)	0.697		
***Specimen type***								
Respiratory tracts (Yes = 1, No = 0)	1.174 (0.818–1.684)	0.385			0.581 (0.258–1.308)	0.190		
Blood (Yes = 1, No = 0)	0.683 (0.327–1.428)	0.311			4.061 (1.399–11.790)	0.010	4.069 (1.339–12.365)	0.013
Others (Yes = 1, No = 0)	0.923 (0.614–1.386)	0.699			0.914 (0.281–2.973)	0.881		
β-lactamase (Positive = 1, Negative = 0)^a^	0.985 (0.875–1.109)	0.802			0.936 (0.671–1.304)	0.694		
***Specimen source***								
Outpatient department (Yes = 1, No = 0)	1.141 (0.897–1.451)	0.284			1.543 (0.828–2.876)	0.172		
Emergency room (Yes = 1, No = 0)	0.861 (0.638–1.162)	0.327			0.728 (0.287–1.847)	0.504		
Wards (Yes = 1, No = 0)	0.695 (0.570–0.849)	< 0.001	0.816 (0.648–1.029)	0.086	0.380 (0.198–0.728)	0.004	0.517 (0.245–1.091)	0.083
Intensive care units (Yes = 1, No = 0)	1.541 (1.240–1.915)	< 0.001	1.410 (1.094–1.818)	0.008	2.165 (1.233–3.802)	0.007	1.695 (0.880–3.265)	0.115
***Infection route***								
Hospital-acquired infection (Yes = 1, No = 0)	1.048 (0.789–1.392)	0.747			0.394 (0.122–1.274)	0.120		

Multiple drug resistance is defined as acquired non-susceptibility to at least one agent in three or more antimicrobial categories. Extensive drug resistance is defined as susceptible to only one antimicrobial category. Specimens other than those from respiratory tracts and blood include wound, pus, abscess, body fluids, and tissues. Hospital-acquired infection is defined as an infection occurs after 7 days of hospital admission. ^a^*n* = 933. *Abbreviation*: *CI* confidence intervals

## Discussion

A high prevalence of certain drug-resistant bacterial species, promoted by antibiotic overuse within medical communities and patient therapeutic incompliance, has been noticed in Taiwan [12, 22–24]. MDR and XDR are often used to refer to *Staphylococcus aureus*, *Enterococcus* species, *Enterobacteriaceae* (other than *Salmonella* and *Shigella*), *Pseudomonas aeruginosa*, and *Acinetobacter* species because of their epidemiological significance, emerging antimicrobial resistance, and the importance of these bacteria within the healthcare system [13]. MDR *H. influenzae* is an aspect that is rarely discussed, and XDR *H. influenzae* has never been mentioned. Here we report the presence of MDR and XDR *H. influenzae* in Taiwan and address their clinical risk factors.

Although the Taiwanese government introduced self-pay *H. influenzae* type b strain vaccination in 2005 and launched nationwide vaccination in infants from 2010, *H. influenzae* remains a critical problem for people without immunization or with invasive infections caused by non-type b or non-typeable strains, which are now the most common causes of invasive *H. influenzae* infection in many countries [25–27]. One of the limitations of the present study is that serotyping of the *H. influenzae* isolates was not available because most of these bacterial isolates were not preserved until the recent approval by the biosafety committee.

The treatment of *H. influenzae* infection relies mainly on β-lactam antibiotics, predominantly ampicillin. However, modifications of penicillin-binding proteins and the spread of plasmids carrying β-lactamase genes, e.g. TEM-1 [28] and ROB-1 [29], among *H. influenzae* or by other bacterial species, have made ampicillin or other first-line β-lactam antibiotics inefficacious against *H. influenzae*. Not surprisingly, the epidemiology shows that rates of *H. influenzae* that were non-susceptible to ampicillin and amoxicillin-clavulanate were high, especially in 2007. A later decline in ampicillin or amoxicillin-clavulanate non-susceptibility might result from the more prudent use of antibiotics [30], a decrease in antibiotic prescribing by general practices [31], or preference for other classes of antimicrobial agents, such as cephalosporins and quinolones. There has been little information on *H. influenzae* strains that are non-susceptible to cefotaxime. One multicenter study by Wang et al. demonstrated that 5.9% of *H. influenzae* isolates from children in China were non-susceptible to cefotaxime [11]. An Iranian meta-analysis of a collection of 43 articles from different databases showed that the prevalence of *H. influenzae* that was non-susceptible to cefotaxime was 22.3% [32]. Cefotaxime resistance in *H. influenzae* is primarily caused by amino acid substitutions N526K, S385T, and L389F and additional substitutions G555E and Y557H in penicillin-binding protein 3 [10, 33, 34]. Our survey shows that the prevalence of cefotaxime-non-susceptible *H. influenzae* in Taiwan from 2007 to 2018 (2.7%) is not as high as those in the above-mentioned reports. Nevertheless, unlike the sporadic cases reported in other countries [35–38], levofloxacin resistance in *H. influenzae* in Taiwan is more severe. A 6-year multicenter study revealed that the non-susceptibility rate of *H. influenzae* to levofloxacin in Taiwan is 12.5%, and all resistant isolates had at least three mutations in the quinolone resistance-determining regions of GyrA and ParC [9]. The good news is that the incidence of levofloxacin-non-susceptible *H. influenzae* reduced from 20.1% in 2016 to 9.2% in 2018.

In our center, 73.2% (41/56) of cefotaxime non-susceptible isolates and 67.8% (200/295) of levofloxacin non-susceptible isolates came from hospitalized patients. Interestingly, *H. influenzae* isolated from respiratory care wards or respiratory care centers were more susceptible to amoxicillin-clavulanate, cefuroxime, and cefotaxime. This could be attributed to the much less exposure to these three drugs owing to different antibiotics being preferred for the relief of severe respiratory infections caused by other bacterial pathogens. Nonetheless, the link between intensive care units and MDR strains shows that enhanced alertness should be exercised in the treatment of patients with severe infections such as meningitis or septicemia that are caused by *H. influenzae* infection.

According to the guideline from the Clinical & Laboratory Standards Institute, azithromycin, clarithromycin, tetracycline, ertapenem, and imipenem are listed as group C antimicrobial agents for *H. influenzae*. Macrolides, tetracycline, or carbapenems are not the primary choice of treatment for *H. influenzae* infection in most medical care institutions in Taiwan, including our center. Therefore they are not being included in our routine drug susceptibility test unless required by special medical circumstances. Our survey provided valuable information despite not including all categories of antimicrobial agents. Among the 557 MDR *H. influenzae* strains, the most common drug non-susceptibility panel was ampicillin, chloramphenicol and trimethoprim-sulfamethoxazole (*n* = 190, 34.1%) and the second most common panel was ampicillin, chloramphenicol, levofloxacin and trimethoprim-sulfamethoxazole (*n* = 83, 14.9%). For XDR *H. influenzae* strains, the most common drug non-susceptibility panel was ampicillin, amoxicillin-clavulanate, cefuroxime, levofloxacin and trimethoprim-sulfamethoxazole (*n* = 12, 23.1%). Of note, eight XDR strains were non-susceptible to all six drug categories tested, suggesting that complex drug-resistant mechanisms might be involved in the development of severely drug-resistant *H. influenzae*. Blood culture was an independent factor for the isolation of XDR *H. influenzae*. More studies are needed to elucidate whether these XDR strains are inherently invasive and then acquire resistance mechanisms or whether XDR confers an invasive ability to these strains.

The outpatient department, which is a pivotal index for monitoring household and community transmissions, contributes to nearly 20% of the XDR *H. influenzae* strains as well. The Division of Infectious Disease and the Antibiotic Stewardship at our center established standard operating procedures for the management of severe drug resistant bacteria; therefore all patients with XDR *H. influenzae* in our study had received proper medication and healthcare and exhibited no signs of relapse. There are currently no signs of XDR *H. influenzae* nosocomial infection or group infection. We will continue to track the incidence of XDR *H. influenzae*.

## Conclusion

This study reported the emergence, epidemiology, risk factors, and treatment regimen of XDR *H. influenzae* infection. The mean incidence of XDR *H. influenzae* infection from 2007 to 2018 was approximately 2.5%. Fortunately, a group infection or nosocomial spread of XDR *H. influenzae* has not been identified yet. Drug susceptibility testing reveals that cefotaxime still has an efficacy against about 80% of XDR *H. influenzae* strains. Greater attention from a public health point of view should be paid to this problem. Additional prophylactic medication strategies are required to prevent the development and spread of XDR *H. influenzae* infection.

## Methods

### Study design

This study was approved by the Institutional Review Board (EMRP-106-062) and performed at E-DA Hospital (Kaohsiung, Taiwan). Data of 2411 laboratory identifications of *H. influenzae* were collected from 2007 to 2018. After excluding related tests from the same patients during a medication management course, 2091 *H. influenzae* isolates from 1436 male and 655 female patients were finally enrolled. Hospital-acquired infection is defined as infection that occurs after 7 days of hospital admission. Twenty-four patients had received the vaccination for infants against the type b strain of *H. influenzae*. Other patients had no anamnesis of *H. influenzae* vaccination. All the patients were treated according to the antibiotic stewardship of E-DA Hospital.

### Laboratory identification and drug-susceptibility tests

*H. influenzae* was identified using Oxoid X + V factor Disc (Thermo Fisher Scientific, Waltham, MA, USA) prior to 2014 and mass spectrometry (VITEK MS, BioMérieux, Marcy-l’Étoile, France) after 2014. Antimicrobial susceptibility tests were performed using BBL Sensi-Disc antimicrobial susceptibility test discs (Becton, Dickinson and Company, Sparks, MD, USA). The following seven antimicrobial agents were included: ampicillin (10 μg), amoxicillin-clavulanate (20/10 μg), chloramphenicol (30 μg), cefotaxime (30 μg), cefuroxime (30 μg), levofloxacin (5 μg), and trimethoprim-sulfamethoxazole (1.25/23.75 μg). Drug susceptibility was evaluated according to the guideline from the Clinical & Laboratory Standards Institute. ETESTs (BioMérieux) were performed on 100 randomly selected *H. influenzae* isolates to confirm the drug susceptibility patterns. BBL Cefinase™ discs (Becton, Dickinson and Company) were used for the rapid detection of β-lactamase activity in 933 *H. influenzae* isolates.

### Definition of drug resistance status

Six antimicrobial categories, including penicillin (ampicillin), β-lactam combination agent (amoxicillin-clavulanate), phenicol (chloramphenicol), cephem (cefotaxime and cefuroxime), fluoroquinolone (levofloxacin), and folate pathway antagonist (trimethoprim-sulfamethoxazole) were classified according to the guideline from the Clinical & Laboratory Standards Institute (Additional file 5: Table S2). No drug resistance is defined as susceptibility to all antimicrobial agents. Single-drug resistance is defined as non-susceptibility to at least one agent in one antimicrobial category. Two-drug resistance is defined as non-susceptibility to at least one agent in two antimicrobial categories. MDR is defined as non-susceptibility to at least one agent in three or four antimicrobial categories. XDR is defined as non-susceptibility to at least one agent in five or six antimicrobial categories.

### Statistical analysis

SPSS 18.0 for Windows was used for all statistical analyses. Nominal variables were compared using Fisher’s exact tests or Pearson Chi-square tests. Continuous variables were compared using Student’s *t*-tests for two independent groups and one-way analysis of variance with Scheffe’s post hoc tests for multiple groups. For the time-point studies, the incidences of drug non-susceptibility in each year were compared with the overall mean value. Stepwise logistic regression analyses were performed to evaluate factors that were associated with MDR or XDR *H. influenzae*. Significance is set at *P* <  0.05 (2-tailed).

## Supplementary information

**Additional file 1 : Figure S1.** Drug non-susceptibility in *Haemophilus influenzae* isolates from different specimen types.

**Additional file 2 : Figure S2.** Association of the demography of patients with drug non-susceptibility in *Haemophilus influenzae* isolates.

**Additional file 3 : Figure S3.** Association of the demography of patients and infection route with the drug resistant status of *Haemophilus influenzae*.

**Additional file 4 : Table S1.** Characteristics of the patients and specimens of extensive drug resistant *Haemophilus influenzae* isolates.

**Additional file 5 : Table S2.** Breakpoints used to determine susceptible, intermediate, and resistant categories for *Haemophilus influenzae* based on CLSI interpretative criteria.

**Additional file 6.** Dataset. Dataset used in this study.

## Data Availability

The data in this study can be seen in Additional file 6: Dataset.

## Abbreviations

AM: ampicillin
AMC: amoxicillin-clavulanate
C: chloramphenicol
CI: confidence intervals
CTX: cefotaxime
CXM: cefuroxime
ER: emergency room
*H. influenzae*: *Haemophilus influenzae*
ICU: intensive care unit
LVX: levofloxacin
MDR: multiple drug-resistance
OPD: outpatient department
OR: odds ratio
SXT: trimethoprim-sulfamethoxazole
XDR: extensive drug-resistance
