# Supplementary material for: Extensively drug-resistant Haemophilus influenzae – emergence, epidemiology, risk factors, and regimen
Source: BMC Microbiol. 2020 Apr 28;20:102. doi: 10.1186/s12866-020-01785-9 (PMC7189504; doi:10.1186/s12866-020-01785-9)
Supplement: Supplementary file 1 — Additional file 1 : Figure S1. Drug non-susceptibility in Haemophilus influenzae isolates from different specimen types. [file 12866_2020_1785_MOESM1_ESM.pptx]

## Slide 1
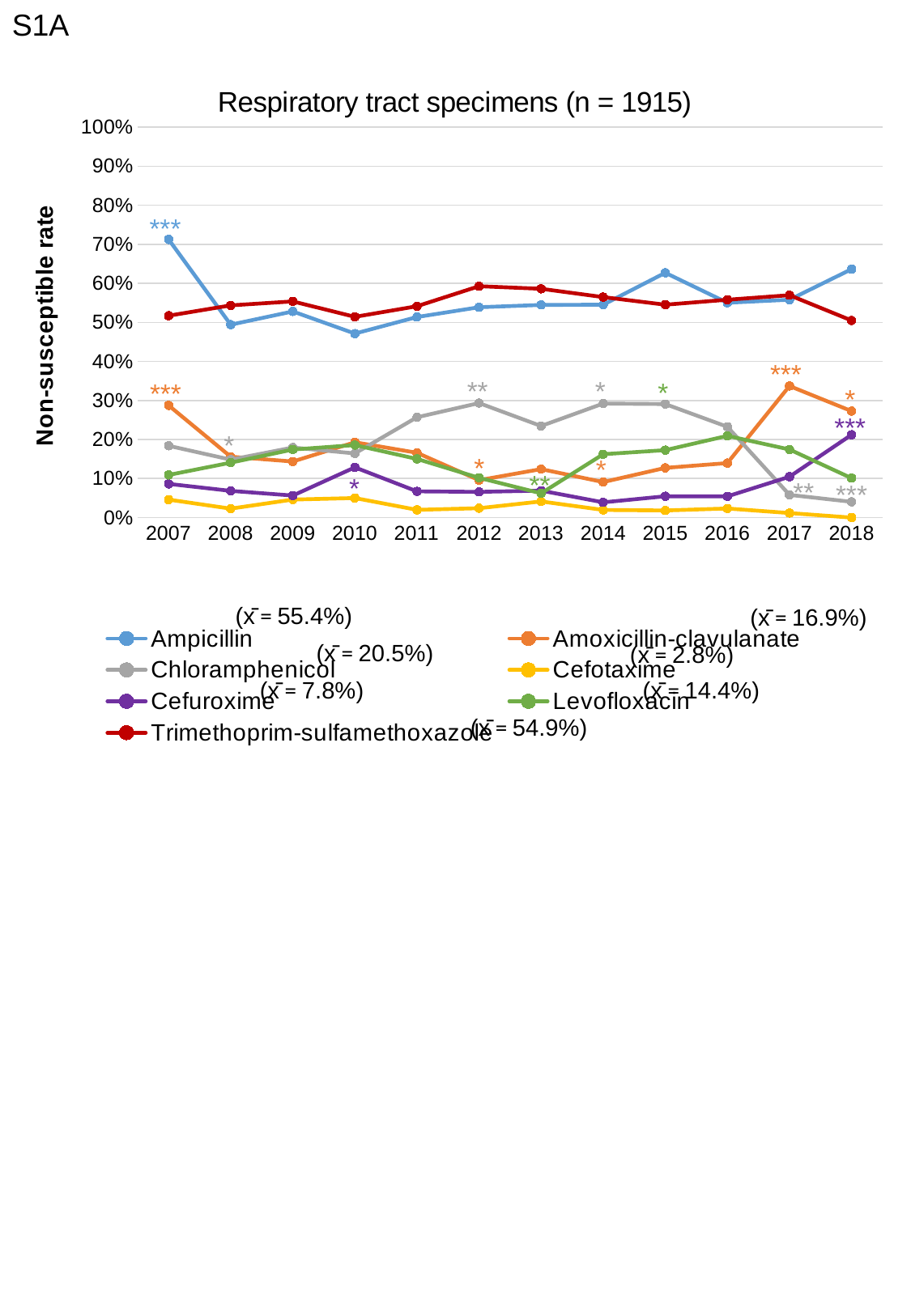

S1A
### Chart: Respiratory tract specimens (n = 1915)
| Category | Ampicillin | Amoxicillin-clavulanate | Chloramphenicol | Cefotaxime | Cefuroxime | Levofloxacin | Trimethoprim-sulfamethoxazole |
|---|---|---|---|---|---|---|---|
| 2007 | 0.7126436781609196 | 0.28735632183908044 | 0.1839080459770115 | 0.04597701149425287 | 0.08620689655172414 | 0.10919540229885058 | 0.5172413793103449 |
| 2008 | 0.49429657794676807 | 0.155893536121673 | 0.1482889733840304 | 0.022813688212927757 | 0.06844106463878327 | 0.14068441064638784 | 0.5437262357414449 |
| 2009 | 0.5282051282051282 | 0.14358974358974358 | 0.1794871794871795 | 0.046153846153846156 | 0.05641025641025641 | 0.17435897435897435 | 0.5538461538461539 |
| 2010 | 0.4714285714285714 | 0.19285714285714287 | 0.16428571428571428 | 0.05 | 0.12857142857142856 | 0.18571428571428572 | 0.5142857142857142 |
| 2011 | 0.5138339920948617 | 0.16600790513833993 | 0.25691699604743085 | 0.019762845849802372 | 0.06719367588932806 | 0.15019762845849802 | 0.541501976284585 |
| 2012 | 0.5389221556886228 | 0.09580838323353294 | 0.2934131736526946 | 0.023952095808383235 | 0.0658682634730539 | 0.10179640718562874 | 0.592814371257485 |
| 2013 | 0.5448275862068965 | 0.12413793103448276 | 0.23448275862068965 | 0.041379310344827586 | 0.06896551724137931 | 0.06206896551724138 | 0.5862068965517241 |
| 2014 | 0.5454545454545454 | 0.09090909090909091 | 0.2922077922077922 | 0.01948051948051948 | 0.03896103896103896 | 0.16233766233766234 | 0.564935064935065 |
| 2015 | 0.6272727272727273 | 0.12727272727272726 | 0.2909090909090909 | 0.01818181818181818 | 0.05454545454545454 | 0.17272727272727273 | 0.5454545454545454 |
| 2016 | 0.5503875968992248 | 0.13953488372093023 | 0.23255813953488372 | 0.023255813953488372 | 0.05426356589147287 | 0.20930232558139536 | 0.5581395348837209 |
| 2017 | 0.5581395348837209 | 0.3372093023255814 | 0.05813953488372093 | 0.011627906976744186 | 0.10465116279069768 | 0.1744186046511628 | 0.5697674418604651 |
| 2018 | 0.6363636363636364 | 0.2727272727272727 | 0.04040404040404041 | 0.0 | 0.21212121212121213 | 0.10101010101010101 | 0.5050505050505051 |***
***
**
*
*
***
*
***
*
*
*
**
*
**
***
(x̄ = 55.4%)
(x̄ = 16.9%)
(x̄ = 20.5%)
(x̄ = 2.8%)
(x̄ = 14.4%)
(x̄ = 7.8%)
(x̄ = 54.9%)

## Slide 2
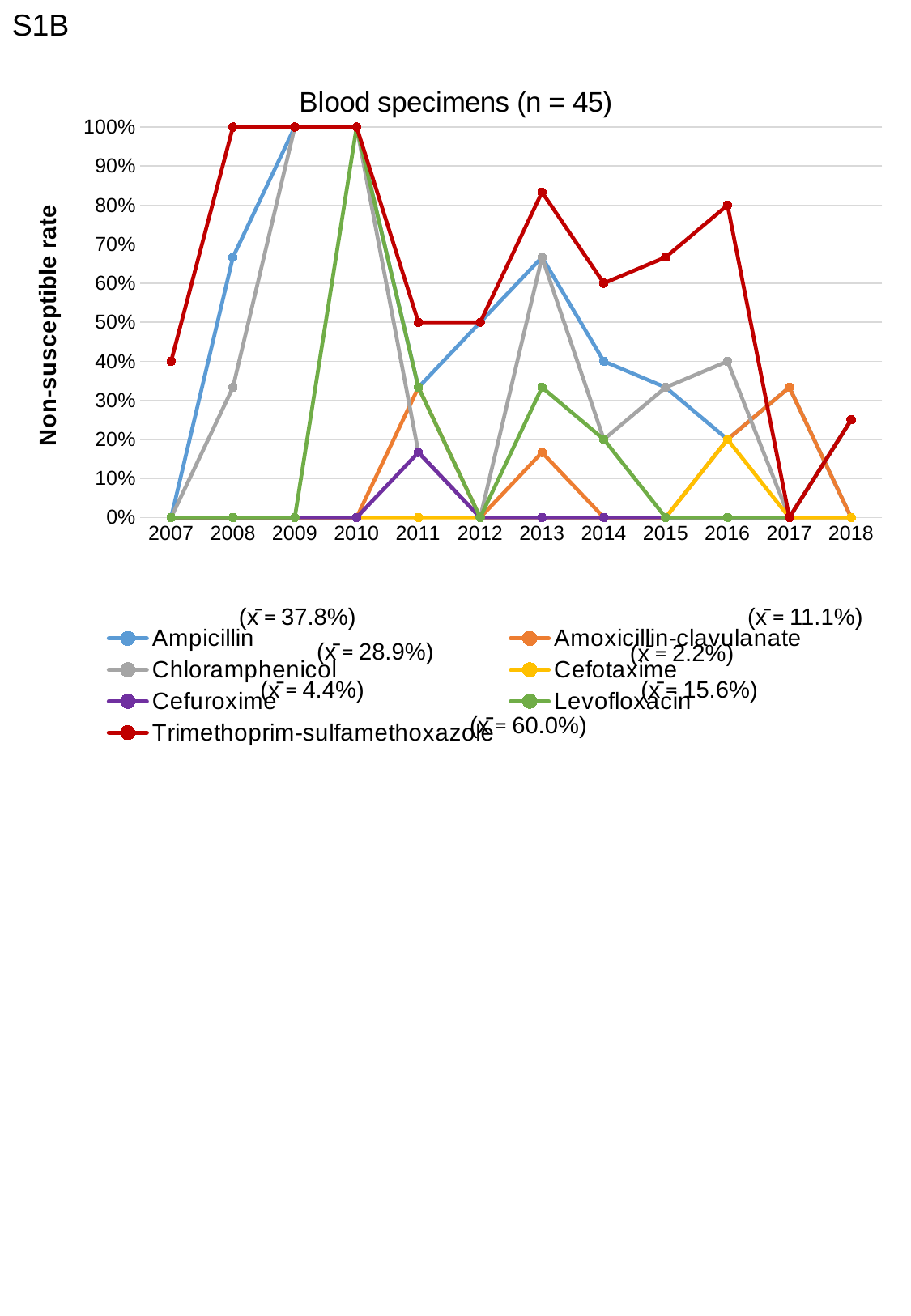

S1B
### Chart: Blood specimens (n = 45)
| Category | Ampicillin | Amoxicillin-clavulanate | Chloramphenicol | Cefotaxime | Cefuroxime | Levofloxacin | Trimethoprim-sulfamethoxazole |
|---|---|---|---|---|---|---|---|
| 2007 | 0.0 | 0.0 | 0.0 | 0.0 | 0.0 | 0.0 | 0.4 |
| 2008 | 0.6666666666666666 | 0.0 | 0.3333333333333333 | 0.0 | 0.0 | 0.0 | 1.0 |
| 2009 | 1.0 | 0.0 | 1.0 | 0.0 | 0.0 | 0.0 | 1.0 |
| 2010 | 1.0 | 0.0 | 1.0 | 0.0 | 0.0 | 1.0 | 1.0 |
| 2011 | 0.3333333333333333 | 0.3333333333333333 | 0.16666666666666666 | 0.0 | 0.16666666666666666 | 0.3333333333333333 | 0.5 |
| 2012 | 0.5 | 0.0 | 0.0 | 0.0 | 0.0 | 0.0 | 0.5 |
| 2013 | 0.6666666666666666 | 0.16666666666666666 | 0.6666666666666666 | 0.0 | 0.0 | 0.3333333333333333 | 0.8333333333333334 |
| 2014 | 0.4 | 0.0 | 0.2 | 0.0 | 0.0 | 0.2 | 0.6 |
| 2015 | 0.3333333333333333 | 0.0 | 0.3333333333333333 | 0.0 | 0.0 | 0.0 | 0.6666666666666666 |
| 2016 | 0.2 | 0.2 | 0.4 | 0.2 | 0.0 | 0.0 | 0.8 |
| 2017 | 0.3333333333333333 | 0.3333333333333333 | 0.0 | 0.0 | 0.0 | 0.0 | 0.0 |
| 2018 | 0.0 | 0.0 | 0.0 | 0.0 | 0.25 | 0.25 | 0.25 |(x̄ = 37.8%)
(x̄ = 11.1%)
(x̄ = 28.9%)
(x̄ = 2.2%)
(x̄ = 15.6%)
(x̄ = 4.4%)
(x̄ = 60.0%)

## Slide 3
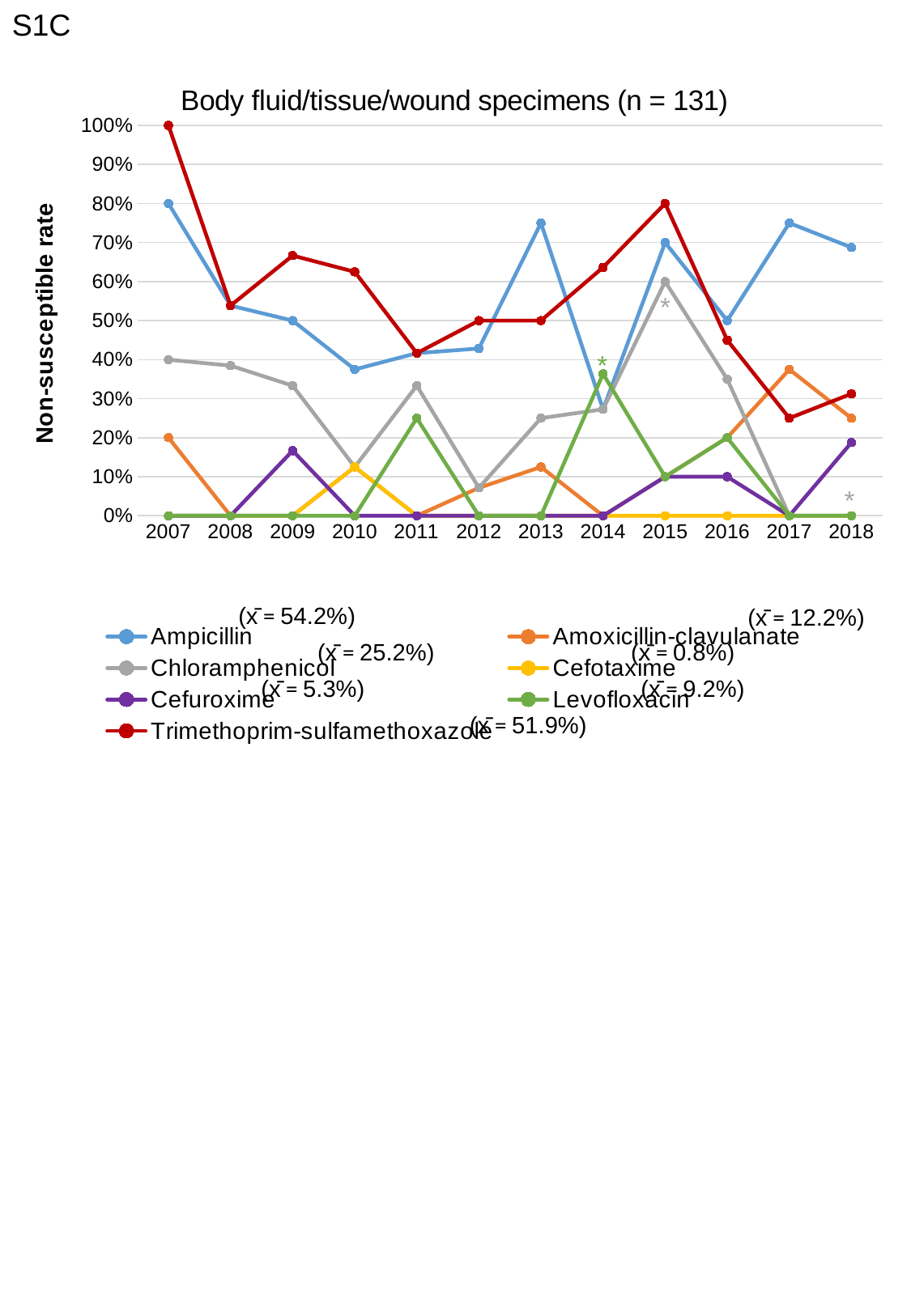

S1C
### Chart: Body fluid/tissue/wound specimens (n = 131)
| Category | Ampicillin | Amoxicillin-clavulanate | Chloramphenicol | Cefotaxime | Cefuroxime | Levofloxacin | Trimethoprim-sulfamethoxazole |
|---|---|---|---|---|---|---|---|
| 2007 | 0.8 | 0.2 | 0.4 | 0.0 | 0.0 | 0.0 | 1.0 |
| 2008 | 0.5384615384615384 | 0.0 | 0.38461538461538464 | 0.0 | 0.0 | 0.0 | 0.5384615384615384 |
| 2009 | 0.5 | 0.0 | 0.3333333333333333 | 0.0 | 0.16666666666666666 | 0.0 | 0.6666666666666666 |
| 2010 | 0.375 | 0.125 | 0.125 | 0.125 | 0.0 | 0.0 | 0.625 |
| 2011 | 0.4166666666666667 | 0.0 | 0.3333333333333333 | 0.0 | 0.0 | 0.25 | 0.4166666666666667 |
| 2012 | 0.42857142857142855 | 0.07142857142857142 | 0.07142857142857142 | 0.0 | 0.0 | 0.0 | 0.5 |
| 2013 | 0.75 | 0.125 | 0.25 | 0.0 | 0.0 | 0.0 | 0.5 |
| 2014 | 0.2727272727272727 | 0.0 | 0.2727272727272727 | 0.0 | 0.0 | 0.36363636363636365 | 0.6363636363636364 |
| 2015 | 0.7 | 0.1 | 0.6 | 0.0 | 0.1 | 0.1 | 0.8 |
| 2016 | 0.5 | 0.2 | 0.35 | 0.0 | 0.1 | 0.2 | 0.45 |
| 2017 | 0.75 | 0.375 | 0.0 | 0.0 | 0.0 | 0.0 | 0.25 |
| 2018 | 0.6875 | 0.25 | 0.0 | 0.0 | 0.1875 | 0.0 | 0.3125 |*
*
*
(x̄ = 54.2%)
(x̄ = 12.2%)
(x̄ = 0.8%)
(x̄ = 25.2%)
(x̄ = 5.3%)
(x̄ = 9.2%)
(x̄ = 51.9%)
