# Supplementary material for: Extensively drug-resistant Haemophilus influenzae – emergence, epidemiology, risk factors, and regimen
Source: BMC Microbiol. 2020 Apr 28;20:102. doi: 10.1186/s12866-020-01785-9 (PMC7189504; doi:10.1186/s12866-020-01785-9)
Supplement: Supplementary file 2 — Additional file 2 : Figure S2. Association of the demography of patients with drug non-susceptibility in Haemophilus influenzae isolates. [file 12866_2020_1785_MOESM2_ESM.pptx]

## Slide 1
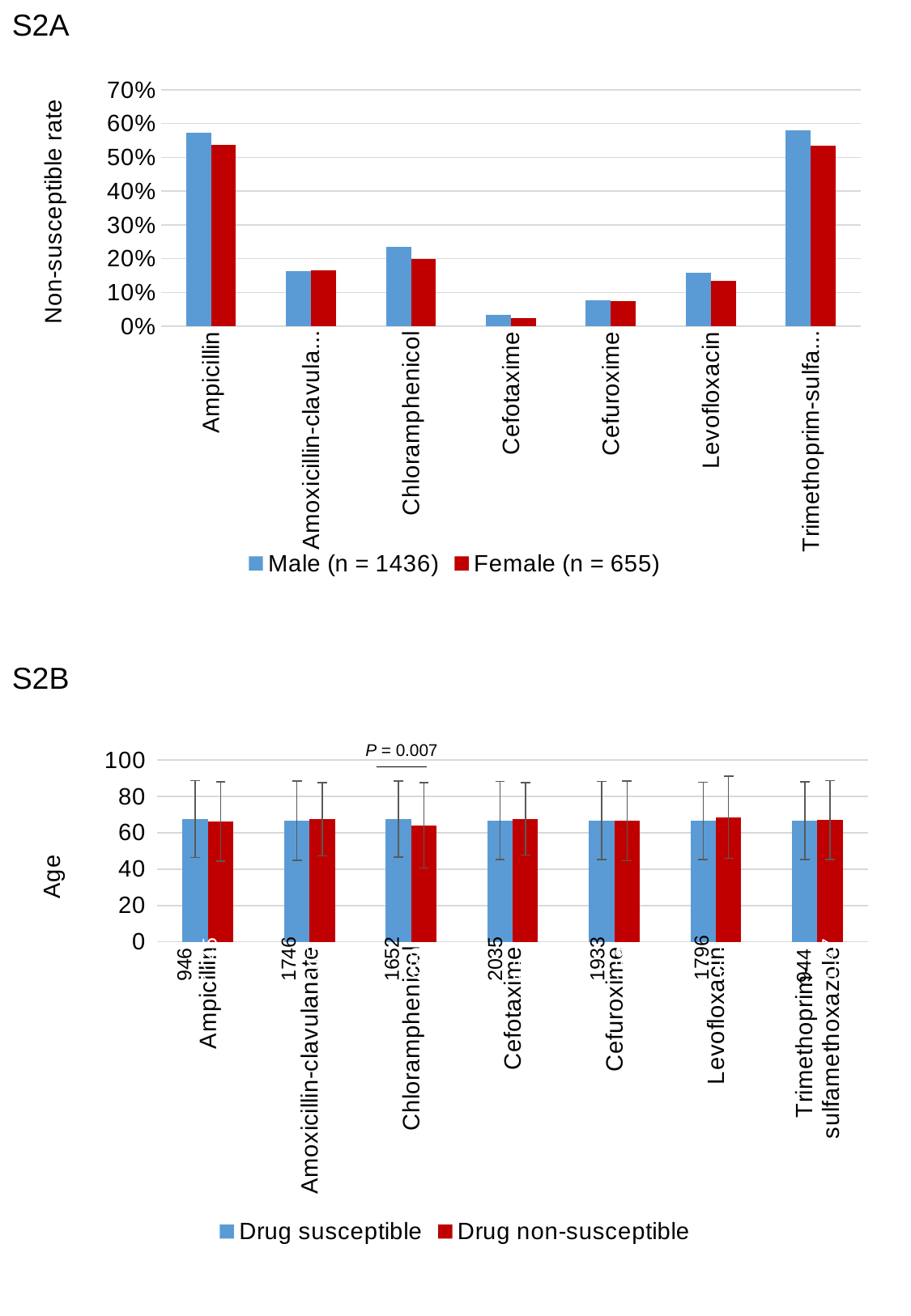

S2A
### Chart
| Category | Male (n = 1436) | Female (n = 655) |
|---|---|---|
| Ampicillin | 0.5725190839694656 | 0.5362116991643454 |
| Amoxicillin-clavulanate | 0.1633587786259542 | 0.16573816155988857 |
| Chloramphenicol | 0.23511450381679388 | 0.19846796657381616 |
| Cefotaxime | 0.03358778625954199 | 0.023676880222841225 |
| Cefuroxime | 0.07633587786259542 | 0.07520891364902507 |
| Levofloxacin | 0.15725190839694655 | 0.13370473537604458 |
| Trimethoprim-sulfamethoxazole | 0.5801526717557252 | 0.5341225626740947 |S2B
P = 0.007
### Chart
| Category | | |
|---|---|---|
| Ampicillin | 67.46440919224955 | 66.2131132379737 |
| Amoxicillin-clavulanate | 66.65091010371005 | 67.42857018049274 |
| Chloramphenicol | 67.47674829487465 | 64.14675986739726 |
| Cefotaxime | 66.75650843118318 | 67.60448385517857 |
| Cefuroxime | 66.80442594021609 | 66.47082538550636 |
| Levofloxacin | 66.50451307315016 | 68.45166194566104 |
| Trimethoprim-sulfamethoxazole | 66.6181220976992 | 66.9118033941761 |295
1796
1145
345
439
56
158
946
1746
1652
2035
1933
1147
944
