# Supplementary material for: Extensively drug-resistant Haemophilus influenzae – emergence, epidemiology, risk factors, and regimen
Source: BMC Microbiol. 2020 Apr 28;20:102. doi: 10.1186/s12866-020-01785-9 (PMC7189504; doi:10.1186/s12866-020-01785-9)
Supplement: Supplementary file 3 — Additional file 3 : Figure S3. Association of the demography of patients and infection route with the drug resistant status of Haemophilus influenzae. [file 12866_2020_1785_MOESM3_ESM.pptx]

## Slide 1
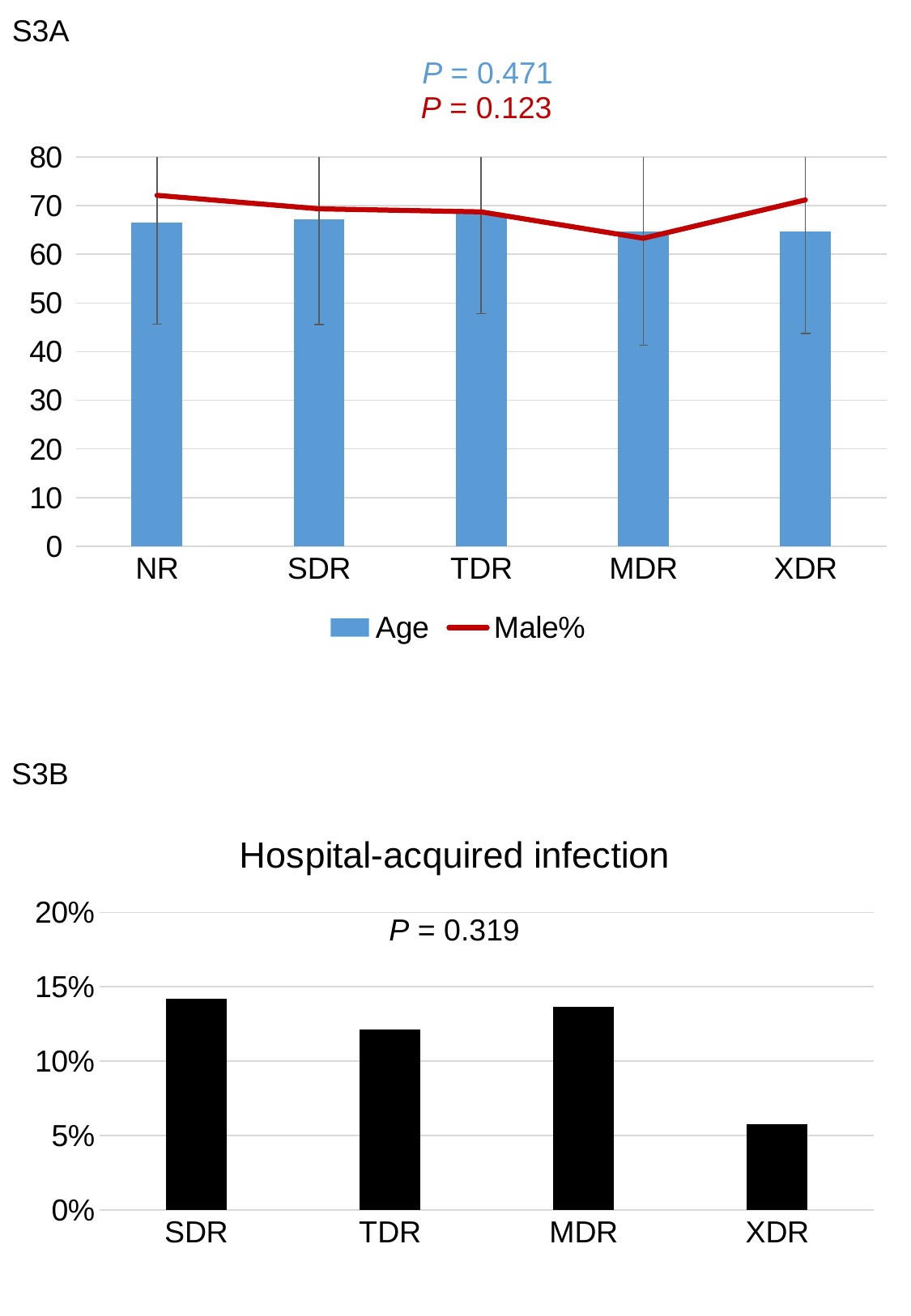

S3A
P = 0.471
P = 0.123
### Chart
| Category | | |
|---|---|---|
| NR | 66.58612567001444 | 72.10144927536231 |
| SDR | 67.17556164382225 | 69.33333333333334 |
| TDR | 68.33054046077812 | 68.70860927152319 |
| MDR | 64.70626625330253 | 63.279445727482674 |
| XDR | 64.64119072673076 | 71.15384615384616 |S3B
### Chart: Hospital-acquired infection
| Category | |
|---|---|
| SDR | 0.14222222222222222 |
| TDR | 0.12108559498956159 |
| MDR | 0.13644524236983843 |
| XDR | 0.057692307692307696 |P = 0.319
