# Supplementary material for: Extensively drug-resistant Haemophilus influenzae – emergence, epidemiology, risk factors, and regimen
Source: BMC Microbiol. 2020 Apr 28;20:102. doi: 10.1186/s12866-020-01785-9 (PMC7189504; doi:10.1186/s12866-020-01785-9)
Supplement: Supplementary file 4 — Additional file 4 : Table S1. Characteristics of the patients and specimens of extensive drug resistant Haemophilus influenzae isolates. [file 12866_2020_1785_MOESM4_ESM.docx]

| **Table S1.** Characteristics of the patients and specimens of extensive drug resistant *Haemophilus influenzae* isolates (n = 52) | | | | | |
| --- | --- | --- | --- | --- | --- |
| No | Year | Age | Specimen type | Specimen source | Drug non-susceptible panel |
| 1 | 2007 | 70's | Sputum | ICU | AM, AMC, CXM, LVX, SXT |
| 2 | 2007 | 60's | Sputum | OPD | AM, AMC, C, CTX, CXM, SXT |
| 3 | 2007 | 60's | Sputum | Ward | AM, AMC, C, CXM, SXT |
| 4 | 2007 | 80's | Sputum | Ward | AM, AMC, C, CXM, SXT |
| 5 | 2007 | 20's | Sputum | ICU | AM, AMC, CTX, CXM, LVX, SXT |
| 6 | 2007 | 70’s | Sputum | OPD | AM, AMC, C, LVX, SXT |
| 7 | 2008 | 80's | Bronchial washing | Ward | AM, AMC, C, LVX, SXT |
| 8 | 2008 | 50's | Sputum | ICU | AM, AMC, CXM, LVX, SXT |
| 9 | 2008 | 80's | Bronchial washing | OPD | AM, AMC, CXM, LVX, SXT |
| 10 | 2009 | 40's | Sputum | OPD | AM, AMC, CTX, LVX, SXT |
| 11 | 2009 | 60's | Sputum | Ward | AM, AMC, C, CXM, LVX, SXT |
| 12 | 2009 | 30's | Sputum | Ward | AM, AMC, C, LVX, SXT |
| 13 | 2009 | 70's | Sputum | ICU | AM, AMC, C, CTX, CXM, SXT |
| 14 | 2010 | 90's | Sputum | OPD | AM, AMC, CTX, CXM, LVX, SXT |
| 15 | 2010 | 90's | Sputum | ICU | AM, AMC, CTX, CXM, LVX, SXT |
| 16 | 2010 | 80's | Sputum | ICU | AM, AMC, CTX, CXM, LVX, SXT |
| 17 | 2010 | 80's | Sputum | ICU | AM, AMC, CXM, LVX, SXT |
| 18 | 2010 | 50's | Sputum | ICU | AM, AMC, CXM, LVX, SXT |
| 19 | 2010 | 50's | Sputum | ICU | AM, AMC, C, CXM, SXT |
| 20 | 2011 | 60's | Blood | ER | AM, AMC, C, LVX, SXT |
| 21 | 2011 | 80's | Sputum | OPD | AM, AMC, C, CTX, CXM, SXT |
| 22 | 2011 | 40's | Sputum | OPD | AM, AMC, C, CXM, LVX, SXT |
| 23 | 2011 | 80's | Sputum | ICU | AM, AMC, C, LVX, SXT |
| 24 | 2011 | 80's | Blood | ER | AM, AMC, CXM, LVX, SXT |
| 25 | 2011 | 10's | Sputum | ICU | AM, AMC, C, CXM, SXT |
| 26 | 2011 | 50's | Sputum | ICU | AM, AMC, CXM, LVX, SXT |
| 27 | 2011 | 50's | Sputum | ICU | AM, AMC, C, CXM, LVX, SXT |
| 28 | 2011 | 10's | Nose inner | OPD | AM, AMC, C, LVX, SXT |
| 29 | 2012 | 60's | Sputum | OPD | AM, C, CXM, LVX, SXT |
| 30 | 2012 | 70's | Sputum | Ward | AM, AMC, C, CXM, LVX, SXT |
| 31 | 2012 | 50's | Sputum | OPD | AM, AMC, C, CXM, LVX, SXT |
| 32 | 2013 | 80's | Sputum | ICU | AM, AMC, C, CXM, SXT |
| 33 | 2013 | 70's | Sputum | ER | AM, AMC, C, CXM, LVX, SXT |
| 34 | 2013 | 60's | Sputum | ICU | AM, AMC, C, CXM, SXT |
| 35 | 2013 | 10's | Nose inner | OPD | AM, AMC, C, CXM, SXT |
| 36 | 2013 | 60's | Blood | ER | AM, AMC, C, LVX, SXT |
| 37 | 2014 | 80's | Sputum | Ward | AM, AMC, C, CXM, SXT |
| 38 | 2014 | 60's | Sputum | Ward | AM, AMC, CXM, LVX, SXT |
| 39 | 2015 | 40's | Wound | ICU | AM, AMC, C, CXM, SXT |
| 40 | 2015 | 60's | Sputum | ICU | AM, AMC, CTX, CXM, LVX, SXT |
| 41 | 2016 | 30's | Wound | OPD | AM, AMC, C, CXM, LVX, SXT |
| 42 | 2016 | 50's | Sputum | ICU | AM, AMC, C, LVX, SXT |
| 43 | 2016 | 50's | Blood | ER | AM, AMC, C, CTX, SXT |
| 44 | 2016 | 70's | Bronchial washing | OPD | AM, AMC, C, LVX, SXT |
| 45 | 2016 | 30's | Wound | OPD | AM, AMC, C, CXM, LVX, SXT |
| 46 | 2016 | 60's | Sputum | ICU | AM, AMC, CXM, LVX, SXT |
| 47 | 2017 | 90's | Sputum | ICU | AM, AMC, CXM, LVX, SXT |
| 48 | 2017 | 70's | Sputum | ICU | AM, AMC, CTX, CXM, LVX, SXT |
| 49 | 2017 | 60's | Sputum | Ward | AM, AMC, CXM, LVX, SXT |
| 50 | 2018 | 70's | Sputum | Ward | AM, AMC, CXM, LVX, SXT |
| 51 | 2018 | 70's | Sputum | Ward | AM, AMC, C, CXM, SXT |
| 52 | 2018 | 60's | Sputum | Ward | AM, AMC, C, CXM, SXT |
| Extensive drug resistance is defined as susceptible to only one antimicrobial category. Abbreviations: AM, ampicillin; AMC, amoxicillin-clavulanate; C, chloramphenicol; CTX, cefotaxime; CXM, cefuroxime; ER, emergency room; ICU, intensive care unit; LVX, levofloxacin; OPD, outpatient department; SXT, trimethoprim-sulfamethoxazole. | | | | | |
