# Supplementary material for: Extensively drug-resistant Haemophilus influenzae – emergence, epidemiology, risk factors, and regimen
Source: BMC Microbiol. 2020 Apr 28;20:102. doi: 10.1186/s12866-020-01785-9 (PMC7189504; doi:10.1186/s12866-020-01785-9)
Supplement: Supplementary file 5 — Additional file 5 : Table S2. Breakpoints used to determine susceptible, intermediate, and resistant categories for Haemophilus influenzae based on CLSI interpretative criteria. [file 12866_2020_1785_MOESM5_ESM.docx]

| **Table S2.** Breakpoints used to determine susceptible, intermediate, and resistant categories for *Haemophilus influenzae* based on CLSI interpretative criteria | | | | | | | | | | | |
| --- | --- | --- | --- | --- | --- | --- | --- | --- | --- | --- | --- |
|  |  |  |  | Interpretive categories and  zone diameter breakpoints,  nearest whole mm | | |  | Interpretive categories and  MIC breakpoints, μg/mL | | | |
| Test/Report group | Antimicrobial agent | Category | Disk content | S | I | R |  | S | I | R | |
| A | Ampicillin | Penicillin | 10 μg | ≥ 22 | 19–21 | ≤ 18 |  | ≤ 1 | 2 | ≥ 4 | |
| C | Amoxicillin-clavulanate | β-lactam combination agent | 20/10 μg | ≥ 20 | – | ≤ 19 |  | ≤ 4/2 | – | ≥ 8/4 | |
| C | Chloramphenicol | Phenicol | 30 μg | ≥ 29 | 26–28 | ≤ 25 |  | ≤ 2 | 4 | ≥ 8 | |
| B | Cefotaxime | Cephem | 30 μg | ≥ 26 | — | — |  | ≤ 2 | — | — | |
| C | Cefuroxime | Cephem | 30 μg | ≥ 20 | 17–19 | ≤ 16 |  | ≤ 4 | 8 | ≥ 16 | |
| B | Levofloxacin | Fluoroquinolone | 5 μg | ≥ 17 | — | — |  | ≤ 2 | — | — | |
| C | Trimethoprim-sulfamethoxazole | Folate pathway antagonist | 0.25/23.75 μg | ≥ 16 | 11–15 | ≤ 10 |  | ≤ 0.5/9.5 | 1/19–2/38 | ≥ 4/76 | |
| Categories of antimicrobial agents are made according to CLSI M100 29^th^ edition. Test/Report groups: Group A, antimicrobial agents are considered appropriate for inclusion in a routine, primary testing panel, as well as for routine reporting of results for the specific organism groups; Group B, antimicrobial agents that may warrant primary testing, but they may be reported only selectively, such as when the organism is resistant to agents of the same antimicrobial class, as in group A. Other indications for reporting the result might include a selected specimen source; a polymicrobial infection; infections involving multiple sites; cases of patient allergy, intolerance, or failure to respond to an antimicrobial agent in group A; or for infection control purposes; Group C, alternative or supplemental antimicrobial agents that may necessitate testing in those institutions that harbor endemic or epidemic strains resistant to several of the primary drugs (especially in the same class, eg, β-lactams); for treatment of patients allergic to primary drugs; for treatment of unusual organisms; or for reporting to infection control as an epidemiological aid. Abbreviations: CLSI, the Clinical & Laboratory Standards Institute;I, intermediate; MIC, minimal inhibitory concentration; R, resistant; S, susceptible. | | | | | | | | | | |  |
